# De Novo SCLC Transformation From *KRAS* G12C-Mutated Lung Adenocarcinoma With Excellent Response to Sotorasib: A Case Report

**DOI:** 10.1016/j.jtocrr.2023.100510

**Published:** 2023-03-29

**Authors:** Takanori Ito, Issei Oi, Zentaro Saito, Takuma Imakita, Osamu Kanai, Kohei Fujita, Hiromasa Tachibana, Koki Moriyoshi, Tadashi Mio

**Affiliations:** a Division of Respiratory Medicine, Center for Respiratory Diseases, National Hospital Organization Kyoto Medical Center, Kyoto, Japan; b Division of Pathology, National Hospital Organisation Kyoto Medical Center, Kyoto, Japan

**Keywords:** *KRAS* G12C, Transformation, Small cell lung cancer (SCLC), Sotorasib, Case report

## Abstract

The transformation to SCLC is a known mechanism of resistance against molecularly targeted therapies. This study reports a patient with untreated lung adenocarcinoma, characterized by a *KRAS* G12C mutation, which transformed into SCLC before treatment. Both the adenocarcinoma and SCLC components were responsive to sotorasib.

## Introduction

Advanced lung cancer has a poor prognosis, but the emergence of molecularly targeted therapies improved the prognosis of eligible patients. The *KRAS* G12C inhibitor sotorasib has been frequently used to treat patients with NSCLC harboring *KRAS* G12C mutations.

The conversion to SCLC is a known mechanism of resistance against molecularly targeted therapies. De novo transformation rarely occurs, and most cases involve patients with *EGFR* mutation-positive lung cancers.^1^ This study reports a patient with untreated lung adenocarcinoma, characterized by a *KRAS* G12C mutation, which transformed into SCLC before treatment. Moreover, both the adenocarcinoma and SCLC components were responsive to sotorasib.

## Case Presentation

**Figure 1:**
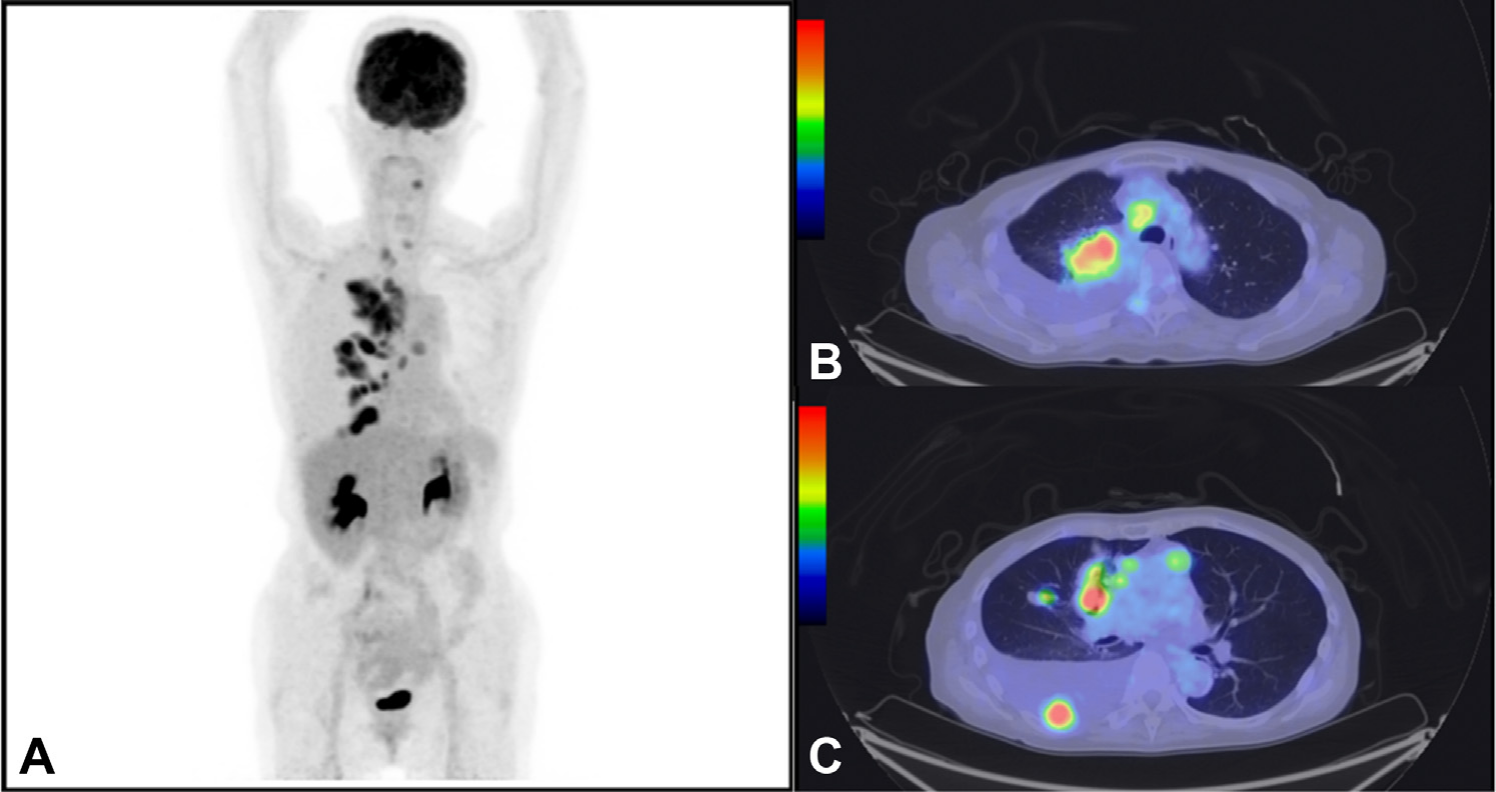
Positron emission tomography-CT on diagnosis. Positron emission tomography-CT revealed multiple lung tumors, pleural effusion, metastatic lesions of mediastinal and neck lymph nodes and bones, and fluorodeoxyglucose accumulation in the same lesions. (*A*) Overall view. (*B*) Tumor at right upper lung and pleural effusion. (*C*) Tumor in the right pleural effusion. CT, computed tomography.

**Figure 2:**
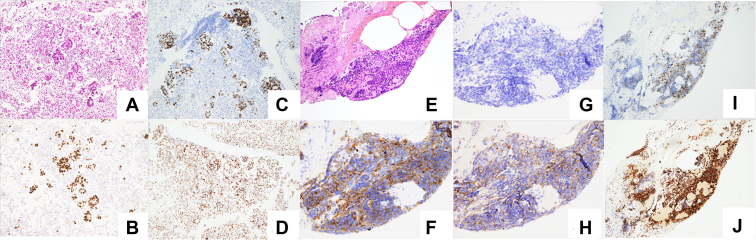
Pathology of the cell block and transbronchial biopsies. Pathology of the cell block from the right pleural effusion is illustrated as (*A*) to (*D*) and that of the transbronchial biopsies from the right upper lung is illustrated as (*E*) to (*J*). In the former, HE and TTF-1 staining revealed adenocarcinoma, but no component of SCLC was identified. In contrast, the latter had a component of SCLC and no adenocarcinoma component. (*A*) HE staining, ×100. (*B*) TTF-1 staining, ×100. (*C*) TP53 staining, ×100. (*D*) RB staining, ×100. (*E*) HE staining, ×100. (*F*) Synaptophysin staining, ×100. (*G*) Chromogranin A staining, ×100. (*H*) CD56 staining, ×100. (*I*) TP53 staining, ×100. (*J*) RB staining, ×100. HE, hematoxylin and eosin.

**Figure 3:**
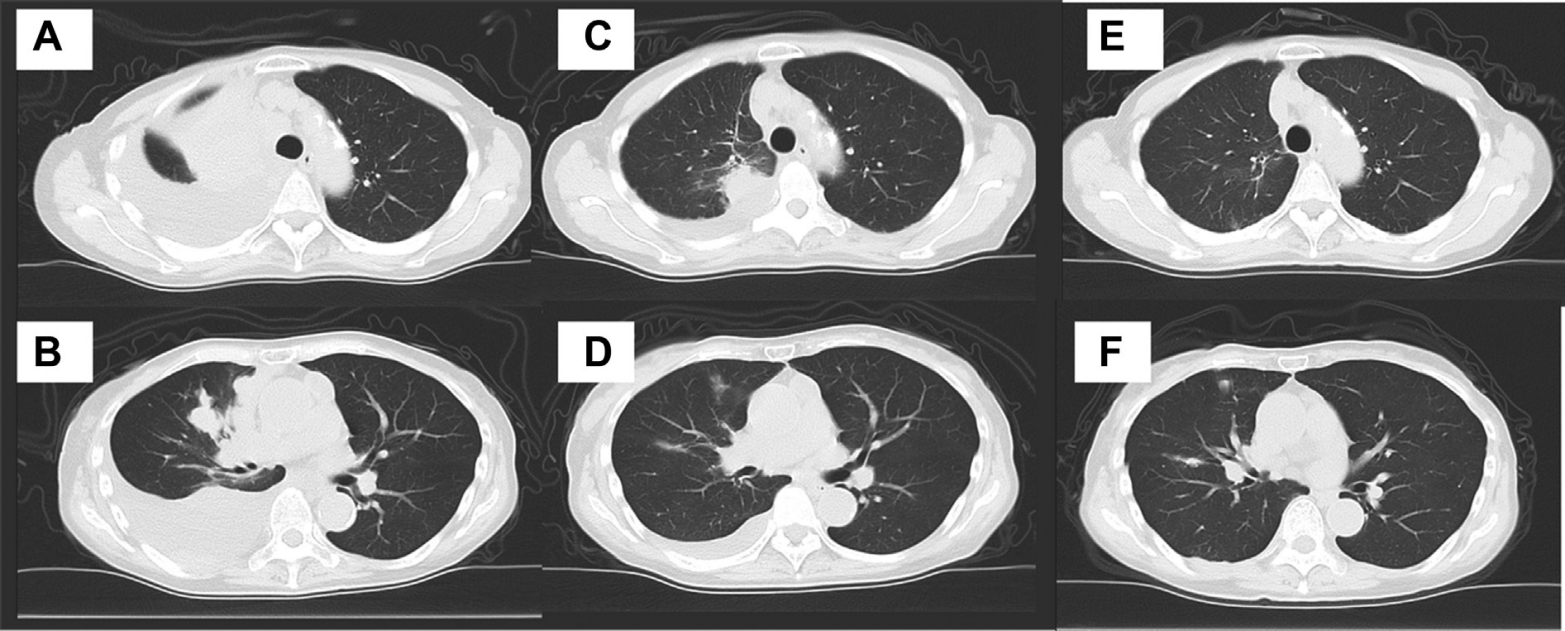
Clinical course based on CT before and after sotorasib. (*A* and *B*) Just before sotorasib administration. (*C* and *D*) One month after sotorasib administration, the tumors had shrunk and pleural effusion had decreased. (*E* and *F*) Four months after sotorasib administration, antitumor effects of sotorasib persist. CT, computed tomography.

A 73-year-old woman with right-sided chest pain and dyspnea was admitted to our hospital. The patient was normotensive and a nonsmoker. Chest radiography result revealed a massive right pleural effusion. In addition, computed tomography revealed multiple nodules in the right lung field, right pleural effusion, and enlarged mediastinal and neck lymph nodes. Positron emission tomography-computed tomography revealed fluorodeoxyglucose accumulation in the tumor region, enlarged mediastinal and neck lymph nodes, multiple sites of bony involvement, and tumors in the region of the right pleural effusion (Fig. 1*A*–*C*). Laboratory workup revealed elevated tumor markers (carcinoembryonic antigen: 79.0 ng/mL, neuron-specific enolase: 41.8 ng/mL, progastrin-releasing peptide: 1200 pg/mL). A right thoracotomy was performed, and transparent yellow pleural fluid was drained. Cytologic examination revealed an adenocarcinoma. A cell block was created using the pleural fluid, and the sample was positive for TTF-1. On this basis, the patient was diagnosed with having lung adenocarcinoma (Fig. 2*A* and *B*). Gene mutation analysis using next-generation sequencing (NGS) (The Oncomine Dx Target Test Multi-CDx System [Ion Torrent PGM Dx Sequencer; Thermo Fisher Scientific]) revealed that the patient harbored a *KRAS* G12C mutation. On Therascreen, the patient was also positive for a *KRAS* G12C mutation. Other gene fusions by NGS are found in Supplementary Data 1 and 2. A bronchoscopy and a transbronchial biopsy of the right upper lung tumor were performed. Pathologic examination revealed SCLC (Fig. 2*E*–*H*), and adenocarcinoma components were not observed. The NGS result for this specimen was remarkable for *KRAS* G12C mutations. TP53 and RB by immunohistochemistry (IHC) were positive in both adenocarcinoma and SCLC components (Fig. 2*C*, *D*, *I*, and *J*). Both SCLC and adenocarcinoma components were detected at different sites. Nevertheless, because metastatic lesions were observed, surgical removal was not feasible. Thus, the patient underwent chemotherapy for SCLC, consisting of carboplatin (area under curve 5) and etoposide (100 mg/m^2^). After two courses, however, the tumor size and carcinoembryonic antigen level increased; therefore, the patient was diagnosed with having a progressive disease. On discussing the treatment options with the patient, sotorasib (960 mg/d) was selected as the second-line treatment. Eight days after the administration of sotorasib, the pleural effusion decreased, and the tumor shrunk. After 3 months, the patient developed mild hepatic dysfunction; therefore, sotorasib was restarted at half the dose after a 1-week withdrawal period to allow normalization of the hepatic function. Four months have passed since the initiation of the second-line therapy, and she still has a good response to the drug (Fig. 3*A*–*F*).

## Discussion

This study reports the de novo transformation to SCLC in a patient with *KRAS* G12C-mutated lung adenocarcinoma. The adenocarcinoma and small cell components responded favorably to sotorasib, a *KRAS* inhibitor.

The transformation into SCLC was first reported by Zakowski et al.^2^ in 2006. On the basis of the subsequent reports on this disease, it occurs in 3% to 14% of cases.^3^^,^^4^ Most cases have resistance to EGFR tyrosine kinase inhibitors (TKIs), and de novo transformation rarely occurs.^1^ Although the mechanism remains unclear, histologic differentiation from adenocarcinoma to SCLC reportedly occurred. This hypothesis was supported by the presence of the same EGFR mutations after the transformation.^3^ In the present case, the patient had an SCLC component before treatment, and similar genetic expressions by NGS were observed in the SCLC and adenocarcinoma components, suggesting histologic differentiation. Biomarkers to predict SCLC transformation are not identified, but current evidence supports double-positive *TP53* and *RB* mutations as potential predictors of SCLC transformation in EGFR-mutated NSCLC.^5^ Moreover, *MYC* amplification was also thought to function as an oncogenic driver in a subset of small cell tumors.^6^^,^^7^ Although our NGS could not examine *TP53* and *RB* mutations, the tumor turned out positive for *MYC* in both its adenocarcinoma and SCLC components. In the IHC, both adenocarcinoma and SCLC components were also positive for RB. In four of five previously reported cases of adenocarcinoma and de novo SCLC transformation, IHC staining revealed the same result for both (i.e., either both were positive or both were negative).^8^, ^9^, ^10^ Although many cases of conversion from positive in adenocarcinoma to negative in SCLC have been reported in resistant cases post-TKIs,^11^ there was no case wherein both were positive. Positivity of RB in IHC indicates a functional RB pathway,^12^ and our results strongly support the concept that both tumor components of de novo SCLC and adenocarcinoma share a common histologic origin. Because this is the first case in which IHC for both adenocarcinoma and SCLC with *KRAS* mutation was performed, further cases are needed to determine whether it is specific to this case.

The treatment strategy for mutated lung adenocarcinoma, which transforms into SCLC, has not been established. Although all SCLC cases with *EGFR* mutations retain the original *EGFR* gene mutation of adenocarcinoma before transformation,^3^ EGFR TKIs are rarely effective against *EGFR* mutation-positive SCLC.^13^ In contrast, the effectiveness of sotorasib in patients with *KRAS* G12C-positive SCLC remains controversial. This case revealed that sotorasib has benefits for patients with *KRAS* G12C mutation-positive SCLC. Furthermore, nonsmoker patients diagnosed with having SCLC should undergo genetic analysis to search for any mutations.

This case report was limited because the possibility of a combined adenocarcinoma and SCLC was not excluded through bronchoscopy biopsy owing to the small volume of the specimen, and adenocarcinoma components were not detected in the specimen with SCLC components. This was an internal medical limitation. Nevertheless, the imaging findings indicated that the SCLC component was also responsive to the treatment.

## Conclusions

This study reported a case of de novo SCLC transformation from *KRAS* G12C-mutated lung adenocarcinoma with a favorable response to sotorasib. This case revealed that molecularly targeted therapeutics was effective against *KRAS* G12C mutation-positive SCLC.

## CRediT Authorship Contribution Statement

**Issei Oi:** Conceptualization, Methodology, Writing—original draft, Review and editing, Supervision, Project administration.

**Takanori Ito:** Writing—original draft, Investigation, Visualizing, Data curation.

**Zentaro Saito, Takuma Imakita, Osamu Kanai, Kohei Fujita, and Hiromasa Tachibana:** Writing—review and editing, Investigation.

**Koki Moriyoshi:** Writing—review and editing, Investigation, Validation.

**Tadashi Mio:** Funding acquisition, Supervision.
