# Supplementary material for: De Novo SCLC Transformation From KRAS G12C-Mutated Lung Adenocarcinoma With Excellent Response to Sotorasib: A Case Report
Source: JTO Clin Res Rep. 2023 Mar 29;4(5):100510. doi: 10.1016/j.jtocrr.2023.100510 (PMC10149245; doi:10.1016/j.jtocrr.2023.100510)
Supplement: Supplementary Data [file mmc1.docx]

Supplementary Data 1. Detected fusion genes by NGS in adenocarcinoma component

| Detected fusion genes (RNA) | read number |
| --- | --- |
| ITGB7 | 2399 |
| LBP1 | 5342 |
| MYC | 224 |
| TBP | 8485 |
| Detected sequence variants (DNA) |  |
| KRAS p.Gly12Cys | Positive |

Supplementary Data 2. Detected fusion genes by NGS in SCLC component

| Detected fusion genes (RNA) | read number |
| --- | --- |
| HMBS | 36 |
| ITGB7 | 4347 |
| LBP1 | 13047 |
| MYC | 433 |
| TBP | 22051 |
| Detected sequence variants (DNA) |  |
| KRAS p.Gly12Cys | Positive |
